# Programmable graphene nanobubbles with three-fold symmetric pseudo-magnetic fields

**DOI:** 10.1038/s41467-019-11038-7

**Published:** 2019-07-16

**Authors:** Pengfei Jia, Wenjing Chen, Jiabin Qiao, Miao Zhang, Xiaohu Zheng, Zhongying Xue, Rongda Liang, Chuanshan Tian, Lin He, Zengfeng Di, Xi Wang

**Affiliations:** 1 State Key Laboratory of Functional Materials for Informatics, Shanghai Institute of Microsystem and Information Technology, Chinese Academy of Sciences, 200050 Shanghai, China; 2 Center of Materials Science and Optoelectronics Engineering, University of Chinese Academy of Sciences, 100049 Beijing, China; 3 The Center for Advanced Quantum Studies, Department of Physics, Beijing Normal University, 100875 Beijing, China; 4 International Center for Quantum Materials, Peking University, 100871 Beijing, China; 5 Department of Physics, State Key Laboratory of Surface Physics and Key Laboratory of Micro- and Nano-Photonic Structure (MOE), Fudan University, 200433 Shanghai, China; 6 Collaborative Innovation Center of Advanced Microstructures, 210093 Nanjing, China

**Keywords:** Condensed-matter physics, Electronic properties and devices

## Abstract

Graphene nanobubbles (GNBs) have attracted much attention due to the ability to generate large pseudo-magnetic fields unattainable by ordinary laboratory magnets. However, GNBs are always randomly produced by the reported protocols, therefore, their size and location are difficult to manipulate, which restricts their potential applications. Here, using the functional atomic force microscopy (AFM), we demonstrate the ability to form programmable GNBs. The precision of AFM facilitates the location definition of GNBs, and their size and shape are tuned by the stimulus bias of AFM tip. With tuning the tip voltage, the bubble contour can gradually transit from parabolic to Gaussian profile. Moreover, the unique three-fold symmetric pseudo-magnetic field pattern with monotonous regularity, which is only theoretically predicted previously, is directly observed in the GNB with an approximately parabolic profile. Our study may provide an opportunity to study high magnetic field regimes with the designed periodicity in two dimensional materials.

## Abstract (web summary)

Presence of nano-bubbles within an atomically thin material generates potentially large pseudo-magnetic fields. Here, the authors report an innovative technique to induce nano-bubbles in graphene with desirable features and high precision through energized AFM tips, and experimentally measure three-fold symmetric pseudo-magnetic fields up to 120 T.

## Introduction

Strain engineering in graphene has been proven as an effective method for modifying its electronic structure due to the remarkable feature of Dirac fermions, such as bandgap opening and pseudo-magnetic fields (PMFs) generation^1^. The introduced strain can induce local changes of Dirac point position at low-energy, and always together with lattice distortions that will change the electron hopping in graphene. That phenomenon can be equivalence of gauge field imitating the electron behavior in real magnetic field, unveiling the signatures of Landau quantization^2,3^. The strain-induced PMFs conserve the time-reversal symmetry unlike real magnetic field and have opposite signs in the K and K’ valleys of graphene forming the low-energy electronic band structure^4^. In consequence, the gauge field can be used as a building block for valleytronic device^5^ or the realization of the Aharonov–Bohm effect by building STM interferometer in strained graphene^6^. The creation of graphene nanobubbles (GNBs) has been proven as an efficient strategy for strain engineering of graphene, thus generating the enormous PMFs^7,8^. For instance, the unconsciously introduced GNBs with the triangular shape on a Pt(111) surface exhibit large uniform PMFs up to 300 T at room temperature^7^, which was not ever obtained previously. Diverse methods had been proposed to create graphene nanobubbles including gas ion irradiation^7–11^, electric field stimulus^12–14^, water splitting^15^, or intense laser irradiation^16^. However, all proposed methods have the limited capability in the manipulation of GNBs including position definition, size control, and shape design, thus greatly impeding the exploration of the relevant physics phenomenon and the practical application of GNBs, though the enormous PMFs exist.

Here, rendered by the accuracy of AFM, the programmable GNBs with the tunable size and shape have been successfully obtained on germanium substrate at the pre-defined locations. With controlling the voltage applied on AFM tip, the contour of bubbles can gradually transform from parabolic profile to Gaussian profile, and the diameter of bubbles can be tuned from tens to hundreds of nanometers. Moreover, higher-resolution scanning tunneling microscopy/spectroscopy (STM/S) reveal that the strain-induced three-fold symmetric pseudo-magnetic field with monotonous regularity exists in GNB with approximately parabolic shape, which is only theoretically predicted previously.

## Results

### Programmable GNBs created by AFM tip

**Fig. 1:**
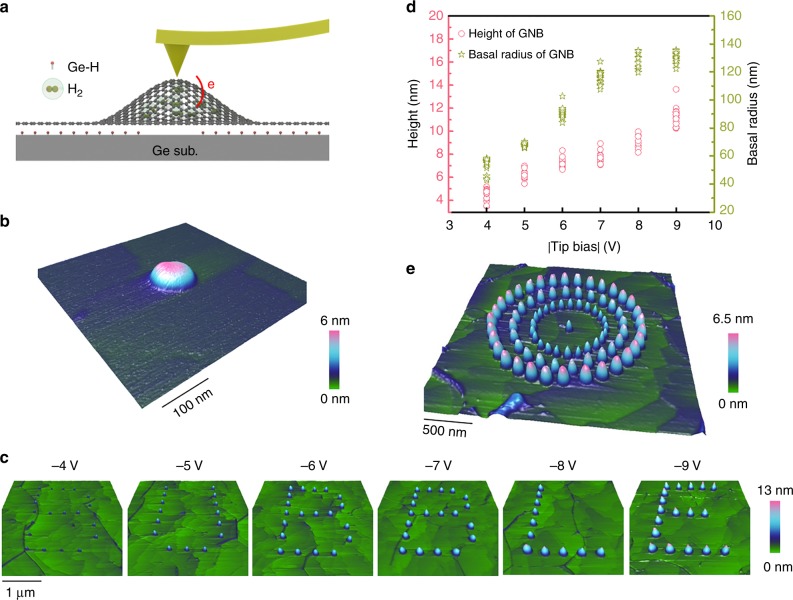
GNBs induced by AFM tip. **a** Schematic illustration of GNBs induced by AFM tip. **b** A GNB induced by AFM tip with the tip voltage of −5 V. **c** BUBBLE pattern of GNBs created by AFM tip with the voltage from −4 to −9 V scanning at the contact mode. **d** Height and basal radius extracted from AFM 3D images in **c** as a function of the tip bias. Source data are provided as a Source Data file. **e** Corral pattern of GNBs created by AFM tip working at the ramp mode. The tip voltages applied to create GNBs located at inner, middle, and outer loops are −6 V, −7 V, and −8 V, respectively

Facilitated by the energized AFM tips, GNBs with the expected size and shape are produced on the monolayer graphene grown on an intrinsic Ge(110) surface (see Methods), as shown in Fig. 1. It is known that hydrogen terminated surface is usually formed when graphene is grown on a clean Ge(110) surface by chemical vapor deposition^17^, as characterized by phase-sensitive sum-frequency vibrational spectroscopy (see Supplementary Note 1 and Supplementary Fig. 1). Due to the low Ge–H bond energy^18^, the hydrogen atoms desorb easily as a local stimulus with negative bias is applied by AFM tip, then evolve into hydrogen molecules^19,20^. Enveloped by the impetrative monolayer graphene, GNBs filled with the released hydrogen molecules will form subsequently as schematically illustrated in Fig. 1a and confirmed by pressure test (see Supplementary Note 2 and Supplementary Fig. 2). Induced by AFM tip with −5 V bias, a typical GNB with ~50 nm basal radius and ~6 nm height is presented in Fig. 1b. It should be noted that the GNB starts to form at −4 V, which corresponds to the low threshold voltage for GNB formation. Our further experiment shows that the cracking GNBs are always formed at the tip bias above −12 V due to the excessive hydrogen molecules enveloped, corresponding to the high threshold voltage for the formation of GNBs (Supplementary Fig. 3). For a positive tip bias, hydrogen atoms are unable to be desorbed, therefore, no GNBs can be formed (Supplementary Fig. 4). In addition, the morphologies of GNBs are less influenced by the energized time and pressure (Supplementary Fig. 5), which is different from laser-induced thermal desorption^21,22^ and oxidation nanolithography^23,24^. The superior maneuverability of AFM tip provides endless possibilities to achieve programmable array of bubbles using a series of preset coordinates. Figure 1c shows a graceful BUBBLE pattern assembled by GNBs with different height and radius obtained by AFM working under the contact mode (see Supplementary Note 3 and Supplementary Movie 1 for detail). As summarized in Fig. 1d, when the tip bias changes from −4 to −9 V, the height and radius of bubbles increase accordingly. In addition to the contact mode, the AFM tip operated under the ramp mode provides more agility to achieve programmable array of GNBs automatically. By pre-setting the tip coordinates, the tip bias and the ramp parameters, the formation of graphene bubbles with the designed pattern will be formed under the guidance of the automatic AFM tip (see Supplementary Note 4 and Supplementary Movie 2 for detail). For instance, the complicate corral pattern, which is well known for the manipulation of iron atoms on a copper surface^25^, can be duplicated by the assembly of GNBs created by automatic AFM tip as well (Fig. 1e, see Supplementary Note 5 and Supplementary Fig. 6). Besides, by multiple scans in the pre-defined area, other than circular GNBs, both linear and rectangular GNBs can be created (Supplementary Note 6 and Supplementary Fig. 7).

### The contour of Programmable GNBs

**Fig. 2:**
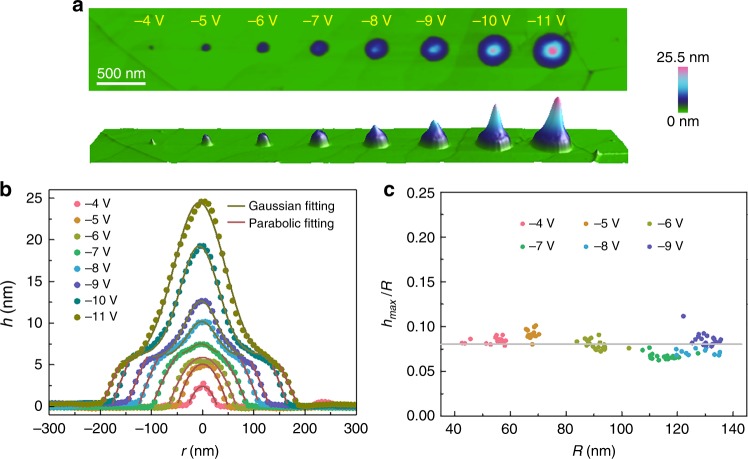
The morphologies of graphene nanobubbles. **a** A series of GNBs created by the tip voltage varying from −4 to −11 V. **b** Line profiles extracted from bubbles in a with parabolic fitting and Gaussian fitting. **c** Measured aspect ratios of GNBs as a function of the base radius. The gray line shows the mean value at 0.08. Source data are provided as a Source Data file

In addition to the tuning of height and radius of GNBs, their contour can also be customized by the applied voltage, thus further regulating the strain distribution of GNBs^26–28^. Figure 2a shows a series of GNBs with the height increment from 2.5 to 25 nm corresponding to the tip bias changing from −4 to −11 V. Besides the increment of height, a gradual transition of GNB profile is also observed, as shown in Fig. 2b (see Supplementary Note 7 and Supplementary Fig. 8). The contour of small GNBs (*R* < 50 nm) can be well fitted by a parabolic curve written as

1 $$z(r) = h_{\max }\left( {1 - \frac{{r^2}}{{R^2}}} \right)$$

where *h*_max_ is the maximum height of bubble and *R* is the radius of bubble base. Our fitting results agree well with the membrane model proposed by Yue et al.^29^ and the unified power form proposed by Lu et al.^30,31^. However, the large GNBs (*R* > 50 nm) exhibit a more complex profile: the bottom part of the bubble can be still fitted by a parabolic curve, but the top part of the bubble changes into a Gaussian contour. The Gaussian contour part of GNBs can be depicted as

2 $$z(r) = \frac{1}{{\sqrt {2\pi } \sigma }}{\mathrm{exp}}\left( { - \frac{r}{{2\sigma ^2}}} \right)$$

where σ is variance of Gaussian distribution and can be written as $\sigma = \frac{1}{{\sqrt {2\pi } h_{\max }}}$. The morphology of GNB is closely related to the boundary condition at the edge of GNB, since graphene is always tightly clamped at the edge of round base for both small and large GNBs. In membrane model, the boundary condition at the edge is determined by the competition between van der Waals (vdW) force and the internal pressure, and GNB has the parabolic profile together with a clamped edge when bending stiffness can be neglected^10,29^. The bending stiffness can be described by the bending rigidity, *κ*, and the in-plane stiffness is always described by Young’s modulus *Y*. Beyond the characteristic parameter $\iota \approx \sqrt {Y/\kappa } \approx$ 4 Å for graphene^10^, the stiffness is dominated by in-plane stiffness. For the GNBs in our study, the dimension is much larger than 4 Å, so the in-plane stresses contribute the majority of the elastic energies, while the contribution of the out-of-plane bending stiffness can be neglected.

In addition, the shape of bubbles is determined by the adhesion energies, which is related to the vdW force between graphene and the substrate. ref. ^10^ concludes a universal law of *h*_max_/*R* considering the stress $\varepsilon = 0$,

3 $$\frac{{h_{{\mathrm{max}}}}}{R} = \left( {\frac{{\pi \gamma }}{{5cY}}} \right)^{1/4}$$

where *γ* is the adhesion energies between graphene and the germanium, the coefficient *c* ≈ 0.7. Figure 2c shows the aspect ratio *h*_max_/*R* behaves approximately as a constant, 0.08, on a wide range of radius or volume. This value is comparable to the aspect ratio *h*_max_/*R* ≈ 0.11 for circular GNBs (*R* > 50 nm), *h*_max_/*L* ≈ 0.07 for triangular GNBs (1000 nm > *L* > 500 nm) obtained on hBN substrate experimentally^10^, or 0.18 for small GNBs filled with helium gas in the graphene interlayer^11^. The similar aspect ratios *h*_max_/*R* suggest the adhesion energy between graphene and hydrogen terminated Ge surface is quite small^17^. Besides, the transition of GNB from the parabolic profile to the combination of parabolic and Gaussian profile may attribute to the change of vdW interactions, which strongly depend on the distance, *h*, between the graphene and substrate, and could be trivial in experimental scales for large GNB. For small GNB with the height *h*_max_ < 5 nm, the vdW force on the center deflection of GNB may exist though is not comparable to that at the edge of round base of GNB^30,31^. Due to the clamping effect at the bubble edge and the internal hydrogen gas pressure, the GNB with parabolic profile is formed. However, when the height of GNB *h*_max_ > 5 nm, the vdW force on the center deflection of GNB is negligible^32,33^ and the corresponding constriction effect disappears. However, the clamping effect at the bubble edge still exists. Therefore, the profile of central part of large bubble transits from the parabolic profile to Gaussian profile, but the parabolic profile near the edge of round base of GNB preserves, as observed in Fig. 2b.

### The pseudo-magnetic fields in GNB

**Fig. 3:**
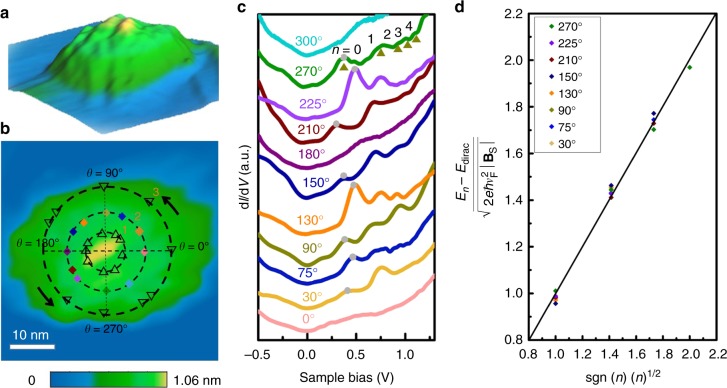
STM and STS of the graphene bubble. **a** 3D STM image (*V*_b_ = −750 mV, *I*_s_ = 0.22 nA) of a typical GNB on Ge(110) with the radius of ~13.5 nm and maximum height of ~1 nm. **b** 2D projection of the GNB shown in **a**. **c** d*I/*d*V* spectra recorded on the colored diamonds at different rotational angles (or second circle line) in **b**. The gray dots indicate the sites of Dirac points. **d** Normalized peak energies $(E_{\it{n}} - E_{{\mathrm{Dirac}}})/\sqrt {2e\hbar v_F^2\left| {{\bf{B}}_S} \right|}$ extracted from the spectra at *θ* = 30°, 75°, 90°, 130°, 150°, 210°, 225°, and 270° in **c** as a function of ${\mathop{\rm{sgn}}} \left( n \right)\left( n \right)^{1/2}$. The linear fitting of Equation (4) is added as well. Source data are provided as a Source Data file

The lattice distortion in GNB creates non-uniform strain distribution, which will modify the low-energy electronic band structure of graphene and change the electron hopping amplitude between carbon atoms, in an equivalent way to the effect of real magnetic fields applied perpendicular to the graphene plane, as known as PMFs^1^. Figure 3a and b show the STM topography of a representative circular graphene bubble on Ge(110) surface induced by the AFM tip with the voltage of −4 V. From the 3D topographic image as shown in Fig. 3a, one could identify an axisymmetric bubble with an approximately parabolic profile of the basal radius ~13.5 nm and the height ~1 nm. Spatially resolved STS is performed to validate the presence of the strain-induced PMFs. Figure 3c shows a series of d*I*/d*V* spectra collected at different rotational angles (denoted as colored diamond points shown in Fig. 3b) along the second circle line counter-clockwisely, and all the spectra exhibit a Dirac-like band structure. It is surprising to observe a series of aperiodic resonances in the tunneling conductance at some peculiar angles, such as 30°, 75°, 90°, 130°, 150°, 210°, 225°, and 270°, while the spectra measured at other angles labeled as 0°, 180°, 300°, exhibit no distinct peaks. The non-equally-spaced resonances eliminate quantum confinement and quasi-bound states as the possible origin of the peaks^1,34–37^. We attribute these peaks to pseudo-Landau levels (pLLs) caused by strain-induced pseudo-magnetic fields^7,14^. In strained graphene, lattice deformation can create PMFs, which affect the behavior of massless Dirac fermions, thus yielding zero-field pLLs quantization. Similar to the influence of a real magnetic field, the PMF gives rise to a sequence of quantized pLLs:

4 $$E_{\it{n}} = \pm \sqrt {2e\hbar v_F^2\left| n \right|\left| {{\bf{B}}_S} \right|} + E_{{\mathrm{Dirac}}}\quad n =\ldots-\!2,\, - 1,\,0,\,1,\,2\ldots$$

Here, *E*_Dirac_ is the energy of Dirac point, e is the electron charge, ℏ is the reduced Planck’s constant, n is the level index, $v_F = 1.0 \times 10^6{\mathrm{m}} \cdot {\mathrm{s}}^{ - 1}$ is the Fermi velocity^38–40^. The positions of the pLLs peaks indexed as *n* = 0, 1, 2, 3, and 4 are marked on Fig. 3c. The normalized peak energy $(E_{\it{n}} - E_{{\mathrm{Dirac}}})/\sqrt {2e\hbar v_F^2\left| {{\bf{B}}_S} \right|}$ collected from eight positions with different rotation angles all exhibit linear dependence on the ${\mathrm{sgn}}\,{\mathrm{(}}n{\mathrm{)}}\,{\mathrm{(}}n{\mathrm{)}}^{1/2}$ as shown in Fig. 3d, which agrees well with the expected scaling behavior described by Equation (4), thus suggesting that the observed peaks originate from pLLs.

## Discussion

**Fig. 4:**
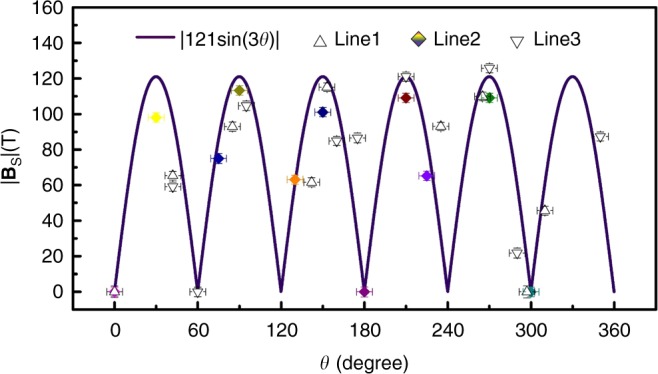
Three-fold symmetric PMF as a function of rotational angles. The experimental |**B**_S_| data extracted from all diamonds and triangles on circle lines 1–3 in Fig. 3b exhibit a six-fold symmetric distribution, which can be well fitted by the function |**B**_S_| = |121sin(3*θ*)|, indicating the presence of three-fold symmetric PMF with alternating signs in GNB. Error bars represent the experimental uncertainties (s.d.) in determining the angles (in *x*-axis) and extracted |**B**_S_| values (in *y* axis). Source data are provided as a Source Data file

It is noticeable that the PMF in the bubble is distributed unevenly and dependent on rotational angles. To illuminate the specific distribution of the PMF, several tens of tunneling spectra (see Fig. 3c, Supplementary Note 8 and Supplementary Figs. 9 and 10) were measured at different angles (denoted as triangles/diamonds) along all three circle lines 1–3 in Fig. 3b, and then the |**B**_S_| data corresponding to each tunneling spectrum can be extracted by linear fitting of the PMFs using Equation (4), as summarized in Fig. 4. It manifests that the absolute values of the |**B**_S_| field distribute from 0 to 125.7 T, where the maximum PMFs is the same order of magnitude to that for graphene nanobubbles randomly formed on Pt(111) surface^7^. Moreover, intriguingly, the maxima of the field occur near the angles of which the interval is nearly 60° at both the edge and the center of GNB, such as 30°, 90°, 150°, 210°, and 270°, and the values diminish and even vanish to zero at the angles between two adjacent ones, such as 60° and 180°. Such the evolution has a six-fold (every 60° in circle lines) symmetry, which can be described by the function |**B**_S_| = |121sin(3*θ*)|. Similar |*B*_*s*_| distribution have also been observed in different GNBs with distinct heights and radii (see more details in Supplementary Note 8 and Supplementary Figs. 11 and 12). We attribute the unusual six-fold symmetric |**B**_S_| field to the PMF with three-fold symmetry due to a sign change between two consecutive maxima, which is only theoretically predicted previously^27,41,42^.

The significant lattice deformation in GNBs could generate an inhomogeneous strain field, which leads to an effective non-uniform PMF. The previous theoretical works have predicted that a rotationally symmetric strain field could be induced in circular GNB and results in a three-fold PMF (or **B**_S_) with alternating signs^14,27,41,42^. Here it should be noted that, the distribution of PMF in our experiment only change as the rotational angle changes, but keep constant for the certain rotational angle even when the distance between the measured position and the bubble edge varies, which is different from the predicted three-fold symmetric PMF occurring only at the bubble edge^27^. The discrepancy may be due to the fact that, unlike the theory prediction, the GNBs with the strictly parabolic shapes could not been obtained in the real experiment, and the configuration complexity of the approximately parabolic GNB including the loss of axisymmetry and the anisotropy of strain may play a crucial role in the formation and modulation of the local PMF distribution. Moreover, GNBs can also be created by AFM tip on other substrates pre-hydrogenated, e.g., SiO_2_ or Si substrate (see Supplementary Note 9 and Supplementary Figs. 13 and 14). As GNBs with the designed size, shape and pattern are easily created by AFM tips, therefore, it is expected that PMFs with the designed distributions can be formed in the graphene on various substrates.

In summary, facilitated by the accuracy of AFM, we present a strategy to realize the programmable GNBs with the designed pattern, and the size and shape of GNB can be tuned by the stimulus voltage applied on AFM tip. Distinct pLLs spectra caused by strain-induced PMFs are observed at GNB by STS measurements, and the PMFs distributed across GNB with an approximately parabolic profile exhibits three-fold symmetry pattern. AFM-facilitated creation of programmable GNBs with enormous pseudo-magnetic fields is expected to provide a unique platform for studying physical phenomena of two dimensional materials under high magnetic field regimes not existing in the reality.

## Methods

### Preparation of graphene film on Ge(110)

Intrinsic Ge(110) wafers (TaiCrystal, >50 ohm.cm, 400 μm thickness) were used in the experiments. The graphene film was synthesized on Ge(110) substrate by chemical vapour deposition in a horizontal tube furnace with H_2_: CH_4_: Ar = 0.7: 23: 220 sccm at the growth temperature of 916 °C for 300 min. Bruker Multimode 8 system was utilized to create the GNBs and measure the morphologies of the GNBs at ambient conditions (temperature ~22 °C, relative humidity ~30%). During the graphene bubbles fabrication, Pt/Ir coated silicon AFM tips with radius of curvature ~30 nm (ANSCM-PC with k = 0.4 N/m, APPNANO) were chosen.

### STM and STS measurement

An ultrahigh vacuum scanning probe microscope (USM-1400S) from UNISOKU was utilized for STM and STS measurements. Both STM and STS measurements were performed in the ultrahigh vacuum chamber (∼10^−11^ Torr) with constant-current scanning mode at liquid-nitrogen temperature of ∼77 K. The STM tips were obtained by chemical etching from a wire of Pt_0.80_Ir_0.20_ alloys. Lateral dimensions of the STM images were calibrated using a standard graphene lattice as well as a Si(111)-(7 × 7) lattice, and, the STS spectra were calibrated using Ag(111) surface. The STS spectra, i.e., the d*I/*d*V* curves, were collected with a standard lock-in technique by turning off the feedback circuit and using a 793-Hz 5 mV a.c. modulation of the sample voltage.

## Supplementary information

Supplementary Information

Description of Additional Supplementary Files

Supplementary Movie 1

Supplementary Movie 2

Source Data

## Data Availability

All data that support the findings of this study are available from the corresponding author upon request. In addition, the source data underlying Figs. 1d, 2b, c, 3c, d and 4c and Supplementary Figs. 1, 9a, b, 10a, b, 11c–e and 12c–e are provided as a Source Data file.

## References

[1] Pereira VM, Castro Neto AH (2009). Strain engineering of graphene’s electronic structure. Phys. Rev. Lett..

[2] Vozmediano MAH, Katsnelson MI, Guinea F (2010). Gauge fields in graphene. Phys. Rep..

[3] Guinea F, Geim AK, Katsnelson MI, Novoselov KS (2010). Generating quantizing pseudomagnetic fields by bending graphene ribbons. Phys. Rev. B.

[4] Morpurgo AF, Guinea F (2006). Intervalley scattering, long-range disorder, and effective time-reversal symmetry breaking in graphene. Phys. Rev. Lett..

[5] Settnes M, Power SR, Brandbyge M, Jauho AP (2016). Graphene nanobubbles as valley filters and beam splitters. Phys. Rev. Lett..

[6] de Juan F, Cortijo A, Vozmediano MAH, Cano A (2011). Aharonov–Bohm interferences from local deformations in graphene. Nat. Phys..

[7] Levy N (2010). Strain-induced pseudo-magnetic fields greater than 300 tesla in graphene nanobubbles. Science.

[8] Lu J, Neto AH, Loh KP (2012). Transforming Moire blisters into geometric graphene nano-bubbles. Nat. Commun..

[9] Zamborlini G (2015). Nanobubbles at GPa pressure under graphene. Nano Lett..

[10] Khestanova E, Guinea F, Fumagalli L, Geim AK, Grigorieva IV (2016). Universal shape and pressure inside bubbles appearing in van der Waals heterostructures. Nat. Commun..

[11] Ghorbanfekr-Kalashami H, Vasu KS, Nair RR, Peeters FM, Neek-Amal M (2017). Dependence of the shape of graphene nanobubbles on trapped substance. Nat. Commun..

[12] Georgiou T (2011). Graphene bubbles with controllable curvature. Appl. Phys. Lett..

[13] Mu W, Zhang G, Ou-Yang Z-c (2013). Radius-voltage relation of graphene bubbles controlled by gate voltage. Appl. Phys. Lett..

[14] Klimov NN (2012). Electromechanical properties of graphene drumheads. Science.

[15] An H (2017). Graphene nanobubbles produced by water splitting. Nano Lett..

[16] Bao Q (2015). Graphene nanobubbles: a new optical nonlinear material. Adv. Opt. Mater..

[17] Lee J-H (2014). Wafer-scale growth of single-crystal monolayer graphene on reusable hydrogen-terminated germanium. Science.

[18] Wintterlin J, Avouris P (1994). Scanning tunneling microscopy (STM) studies of the chemical vapor deposition of Ge on Si(111) from Ge hydrides and a comparison with molecular beam epitaxy. J. Chem. Phys..

[19] Shen TC, Wang C, Abeln GC, Tucker JR (1995). Atomic-scale desorption through electronic and vibrational excitation mechanisms. Science.

[20] Scappucci G, Capellini G, Lee WC, Simmons MY (2009). Atomic-scale patterning of hydrogen terminated Ge(001) by scanning tunneling microscopy. Nanotechnology.

[21] Koehler BG, George SM (1991). Laser-induced desorption of H_2_ from Si (111) 7 × 7. Surf. Sci..

[22] Lewis LB, Segall J, Janda KC (1995). Recombinative desorption of hydrogen from the Ge(100)–(2 × 1) surface: a laser-induced desorption study. J. Chem. Phys..

[23] Weng L, Zhang L, Chen YP, Rokhinson LP (2008). Atomic force microscope local oxidation nanolithography of graphene. Appl. Phys. Lett..

[24] Avouris P, Martel R, Hertel T, Sandstrom R (1998). AFM-tip-induced and current-induced local oxidation of silicon and metals. Appl. Phys. A.

[25] Crommie MF, Lutz CP, Eigler DM (1993). Confinement of electrons to quantum corrals on a metal surface. Science.

[26] Guinea F, Katsnelson MI, Geim AK (2009). Energy gaps and a zero-field quantum Hall effect in graphene by strain engineering. Nat. Phys..

[27] Qi Z (2014). Pseudomagnetic fields in graphene nanobubbles of constrained geometry: a molecular dynamics study. Phys. Rev. B.

[28] Koenig SP, Boddeti NG, Dunn ML, Bunch JS (2011). Ultrastrong adhesion of graphene membranes. Nat. Nanotechnol..

[29] Yue K, Gao W, Huang R, Liechti KM (2012). Analytical methods for the mechanics of graphene bubbles. J. Appl. Phys..

[30] Dai Z (2018). Interface-governed deformation of nanobubbles and nanotents formed by two-dimensional materials. Phys. Rev. Lett..

[31] Sanchez DA (2018). Mechanics of spontaneously formed nanoblisters trapped by transferred 2D crystals. Proc. Natl Acad. Sci. USA.

[32] Wang P, Gao W, Cao Z, Liechti KM, Huang R (2013). Numerical analysis of circular graphene bubbles. J. Appl. Mech..

[33] Wang P, Liechti KM, Huang R (2016). Snap transitions of pressurized graphene blisters. J. Appl. Mech..

[34] Zhao Y (2015). Creating and probing electron whispering-gallery modes in graphene. Science.

[35] Lee J (2016). Imaging electrostatically confined Dirac fermions in graphene quantum dots. Nat. Phys..

[36] Gutiérrez C, Brown L, Kim C-J, Park J, Pasupathy AN (2016). Klein tunnelling and electron trapping in nanometre-scale graphene quantum dots. Nat. Phys..

[37] Bai K-K, Qiao J-B, Jiang H, Liu H, He L (2017). Massless Dirac fermions trapping in a quasi-one-dimensional npn junction of a continuous graphene monolayer. Phys. Rev. B.

[38] Miller DL (2009). Observing the quantization of zero mass carriers in graphene. Science.

[39] Li G, Andrei EY (2007). Observation of Landau levels of Dirac fermions in graphite. Nat. Phys..

[40] Yin L-J, Li S-Y, Qiao J-B, Nie J-C, He L (2015). Landau quantization in graphene monolayer, Bernal bilayer, and Bernal trilayer on graphite surface. Phys. Rev. B.

[41] Settnes M, Power SR, Lin J, Petersen DH, Jauho A-P (2015). Patched Green’s function techniques for two-dimensional systems: Electronic behavior of bubbles and perforations in graphene. Phys. Rev. B.

[42] Schneider M, Faria D, Viola Kusminskiy S, Sandler N (2015). Local sublattice symmetry breaking for graphene with a centrosymmetric deformation. Phys. Rev. B.

