# Supplementary material for: Programmable graphene nanobubbles with three-fold symmetric pseudo-magnetic fields
Source: Nat Commun. 2019 Jul 16;10:3127. doi: 10.1038/s41467-019-11038-7 (PMC6635427; doi:10.1038/s41467-019-11038-7)
Supplement: Supplementary file 1 — Supplementary Information [file 41467_2019_11038_MOESM1_ESM.pdf]

Supplementary Information

Programmable graphene nanobubbles with three-fold symmetric pseudo-magnetic fields

Pengfei Jia, Wenjing Chen, Jiabin Qiao, Miao Zhang, Xiaohu Zheng, Zhongying Xue, Rongda Liang, Chuanshan Tian, Lin He*, Zengfeng Di*, Xi Wang

*Corresponding author. E-mail:helin@bnu.edu.cn; zfdi@mail.sim.ac.cn

Supplementary Note 1: Measurement of hydrogen terminated Ge(110) surface.

After the growth of single crystalline graphene, the dangling bonds on Ge(110) substrate are terminated by atomic hydrogen, as reported by Lee *et al*[1]. However, it is hard to detect the monolayer hydrogen atoms by conventional means. Here, we used the phase-sensitive vibrational spectroscopy (PS-SFVS) to verify the presence of hydrogen-terminated surface. PS-SFVS is an effective tool to probe the vibrational structures of interfaces with high surface specificity[2,3]. It is a second-order nonlinear optical process in which mixing of two input beams in a medium generates a coherent output. Several samples are measured by PS-SFVS including bare Ge(110) substrate, Ge(110) substrate with the transferred graphene, and Ge(110) substrates with different coverage of graphene grown by CVD (Supplementary Figure 1). For Ge(110) substrates with different coverage of graphene, a peak near 2000 cm^{-1} appears and the peak intensity increases gradually as the coverage of graphene increases. However, such a peak is never observed on the bare Ge(110) substrate and Ge(110) substrate with the transferred graphene. Therefore, we attribute the peak to

the Ge-H vibrational mode[4,5] originating from the hydrogen-terminated surface between the single crystalline graphene and Ge(110) substrate.

Supplementary Note 2: Pressure test of graphene nanobubbles.

To validate that the graphene nanobubbles (GNBs) are filled with hydrogen gas, the pressure test was performed on the graphene bubbles. The tip of ACCESS-EFM (k = 3.4 N/m) with -5 V tip bias was utilized to create GNBs. After the formation of GNBs, different loads from 23 to 1380 nN were exerted on the tip successively during the whole image scanning, and the direction of scanning was set from top to down. The AFM images are shown in Supplementary Figures 2a-h. It is obvious that the bubble remains intact when the load is 23 nN. However, due to the weak adhesion between graphene and Ge (110) substrate, the gas leakage channel starts to appear when the loading force increases to 115 nN. The gas leakage channel significantly expands when the load increases to 230 nN. As the loading force is further increased to 460nN or above, an additional gas leakage channel is formed. And, other gas leaked channels will appear at larger loading force. Meanwhile, the height of bubble also decreases significantly due to the release of hydrogen gas through the gas leakage channel, as shown in Supplementary Figure 2i.

Supplementary Note 3: The method to create GNBs by AFM tip at contact mode.

Bruker Multimode 8 system was utilized to create the graphene nanobubbles (GNBs) and measure the morphologies of the GNBs at ambient conditions. The formation process for

graphene bubbles was conduct at contact mode with 10 nm scanning size, 1 Hz scanning velocity and 4 nN contact force for several seconds. The voltage applied to the AFM tip was tuned from -4 to -11 V. The detailed procedure to produce GNBs at contact mode is presented below:

(1) Select the contact mode or lateral force mode.

(2) Turn on the tip bias control at expended panel.

(3) Obtain AFM images on the pristine graphene in order to determine the pre-coordinates of bubbles.

(4) Move the tip to the pre-set location, then set the scanning region to 10×10 nm^2 (or smaller in order to reduce the shake of tip), and the scan rate is 1 Hz, deflection setpoint is 0.1 V (corresponding to ～4.3 nN for ANSCM-PC calibrated on sapphire substrate).

(5) Apply the proper negative voltage to the AFM tip (the influence of tip bias had been discussed in the main text). The energize time can be set to several seconds.

(6) Remove the tip bias and move to the next pre-set locations of GNBs, and repeat step (5) until all the GNBs are fabricated.

(7) Set suitable scanning size to obtain AFM image of GNBs.

The complete process is also recorded in Video 1.

Supplementary Note 4: The method to create GNBs by AFM tip at ramp mode.

The ramp mode is designed for force spectroscopy to display force curve data instead of image data. In this mode, the tip will press down to the surface successively with the pre-

set sites and the constant frequency, and the whole process can be accomplished automatically. By energizing AFM tip with a negative bias, AFM operating under the ramp mode can be further expanded to create GNBs automatically with high efficiency. The detailed procedure is presented below:

(1) Select the contact mode or lateral force mode.

(2) Turn on the tip bias control at expanded mode.

(3) Obtain AFM images on the pristine graphene, and open the "Point and Shoot" panel to record the coordinates of AFM image. "Point and Shoot" window can display high speed data capture functions (we can use this function to display the fabrication of GNBs with high speed).

(4) When the capture of the current image is finished, system will turn to ramp mode automatically, and set up the ramp parameters. Ramp size is always set to 300 nm, ramp rate is 1 Hz, trig threshold is 0.2 V, and tip bias is -4~-11 V.

(5) After setting the ramp parameters, the bubbles' coordinates can be defined on the "Point and Shoot" window. With the control of coordinates, the designed pattern can be drawn in the point tab, or the file containing the designed pattern can be loaded directly.

(6) Start to ramp.

(7) After the ramp process, turn to scanning mode to obtain AFM image of GNBs with the designed pattern.

Fig. 1e and Supplementary Figure 6 show the bubbles with the designed "corral" pattern created by ramp mode. The complete process for the formation of bubble array by ramp

mode is also fully recorded in Video 2. The mode provides an efficient method to create GNBs, and hundreds of GNBs can be fabricated in several minutes.

Supplementary Note 5: The preparation of GNBs.

The creation of GNBs has been intensively studied, as provided in Supplementary Figure 3-6.

Supplementary Note 6: GNBs with the designed morphology.

Most of the GNBs reported in other literatures have round or nearly round bases, and a few of them have triangle[6,7], pyramidal[7] or hexagonal bases[8]. The shape of bubble is mostly determined by the crystal orientation of substrate or the relaxation of strain, therefore, it is infeasible to achieve GNBs with the arbitrary morphology. Rendered by the flexibility and the accuracy of AFM, we are able to create bubbles with diverse morphologies including round, linear or square base by controlling the scanning area of energized AFM tip, as shown in Supplementary Figure 7. The length of linear bubbles is 2 μm, the width is 190 nm, and the height is 8 nm. When creating the linear bubbles, we should set the energized AFM tip (with the tip bias of -12 V in Supplementary Figure 7b) to scan along single line repeatedly. The detailed method can refer to Supplementary Section 2. Similarly, if we set the energized AFM tip to scan at a square area up and down, the bubble with the square morphology will be formed, as shown in Supplementary Figure 7c (with the tip bias of -10 V). The side of square bubble is 2 μm, and the average height is ~11.5 nm. Likewise, only

by adjusting the scanning area, we are able to create GNBs with arbitrary shapes we expected.

Supplementary Note 7：Normalized bubble profiles.

We normalize in-plane positions by their radii and re-plot the data in Figure 2b of the main text, as shown in Supplementary Figure 8(a). The comprehensive normalization including the normalization of out-of-plane profiles by their maximum heights and the normalization of in-plane positions by their radii of the bubbles is also conducted, as displayed in Supplementary Figure 8(b). Both figures depict that the profiles of graphene bubbles are parabolic when created by the voltages between -4 V to -6 V. For the bubbles stimulated by the voltages between -8 V and -11 V, the bottom part of the bubble preserves the parabolic shape, which the top part changes into a Gaussian contour. Meanwhile, the graphene bubbles stimulated by the voltage at -7 V experience the transition stage from the pure parabolic shape to the combination of parabolic shape and Gaussian shape.

Supplementary Note 8: STM/STS data from multiple GNBs.

STS data collected at different rotational angle along the inner and outer circles on the GNB shown in Figure 3 in the main text are provided in Supplementary Figure 9-10. STM/STS data from two other GNBs are provided in Supplementary Figure 11-12, as well.

Supplementary Note 9: The GNBs induced by AFM tip on other substrate.

We can also create programmable graphene nanobubbles by AFM tip on other substrate, e.g., SiO_2 or Si substrate. In our manuscript, the H-terminated surface is essential for programmable GNBs formed on Ge(110) substrate. Due to the low Ge-H bond energy, the hydrogen atoms desorb easily as a local stimulus with negative bias is applied by AFM tip, then evolve into hydrogen molecules filled GNBs. Therefore, our approach can be extended to other conventional substrates such as SiO_2 and Si substrate if the substrates are pretreated by hydrogenation and the essential H-terminated surface is formed.

For instance, the hydrogen terminated surface can be formed on Si substrate by immersing into 5% H_2O_2 for 5 min and then into 5% HF for 5 min. After the transfer of graphene to H-terminated Si substrate, GNB can be also created on Si substrate by AFM tip, as shown in Supplementary Figure. 13a-b. As a local stimulus with negative bias of -6 V is exerted, the GNB with ~50 nm radius and ~8 nm height is obtained, as shown in Supplementary Figure. 13c-d.

Meanwhile, hydrogen termination can be also obtain on SiO_2/Si substrate by the dipping into 10% HCl solution for 20 min at 50 °C. As AFM tip with -5 V voltage is applied, the GNB with ~65 nm radius and ~1.3 nm height can be formed, as shown in Supplementary Figure. 14a-b. When the stimulus bias increases to -10 V, the size of GNB (~75 nm radius and ~1.7 nm height) increases as well, as display in Supplementary Figure. 14c-d. Therefore, the method for creating programmable graphene nanobubbles by AFM tip we proposed in not restricted to Ge(110) substrate, and it can be generally applied to other common substrates.

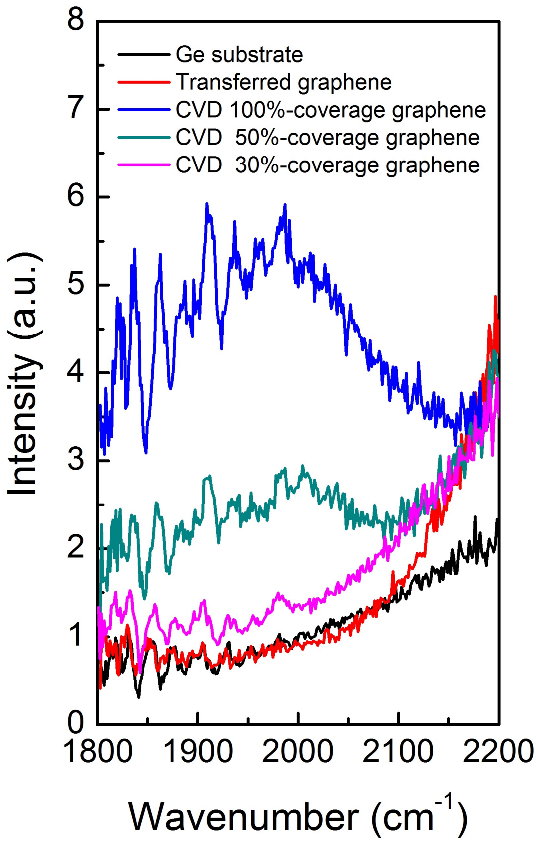

Supplementary Figure 1 | The phase-sensitive sum-frequency vibrational spectroscopy (PS-SFVS) test for verifying the presence of hydrogen terminated surface. PS-SFVS results obtained from bare Ge(110) substrate, Ge(110) substrate with the transferred graphene, and Ge(110) substrates with different coverage of graphene grown by CVD. The peak near 2000 cm^{-1} corresponds to the Ge-H vibrational mode. Source data are provided as a Source Data file.

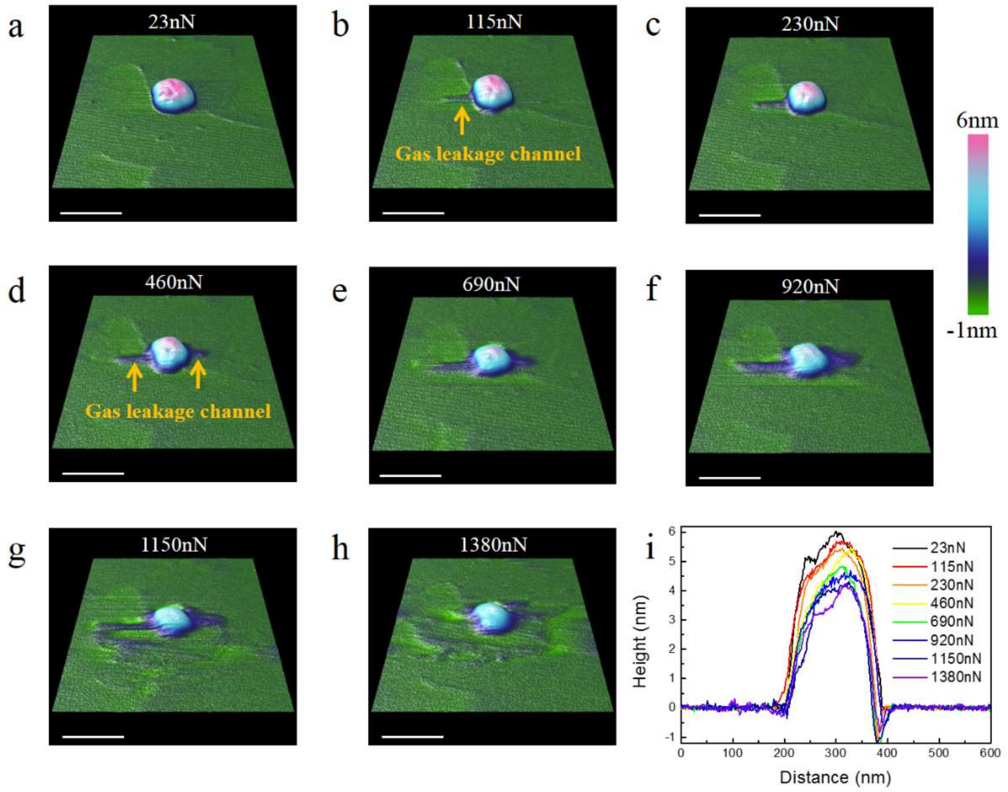

Supplementary Figure 2 | Pressure test of GNBs. The morphology change of GNB pressed by AFM tip with different load. **a**, 23 nN. **b**, 115 nN. **c**, 230 nN. **d**, 460 nN. **e**, 690 nN. **f**, 920 nN. **g**, 1150 nN. **h**, 1380 nN. The orange arrow indicates the gas leakage channel. Scale bar is 200 nm. **i**, The diameter and height of GNB pressed by AFM tip with different load.

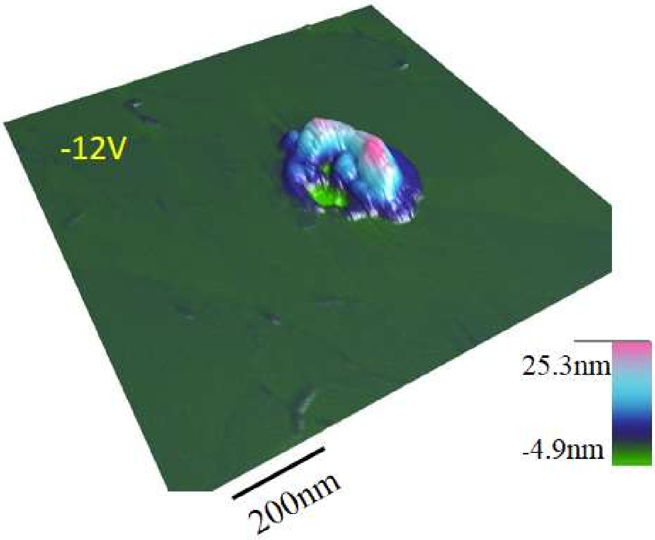

Supplementary Figure 3 | 3D AFM image of the broken graphene nanobubble induced by the tip bias of -12 V.

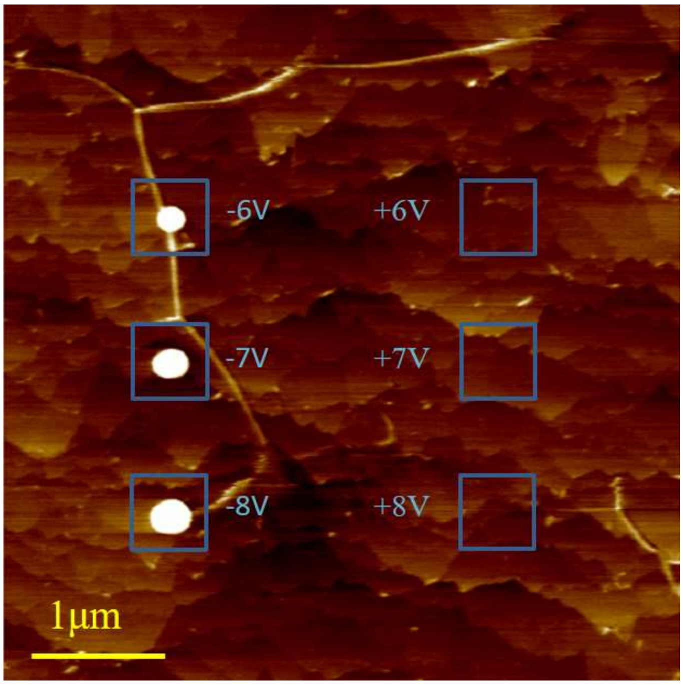

Supplementary Figure 4 | AFM image of the GNBs induced at positive tip bias and negative tip bias. GNBs are formed by the tip with negative bias, and the bubble size increases as the tip bias increases. While, no GNB is formed as the tip bias changes to positive bias.

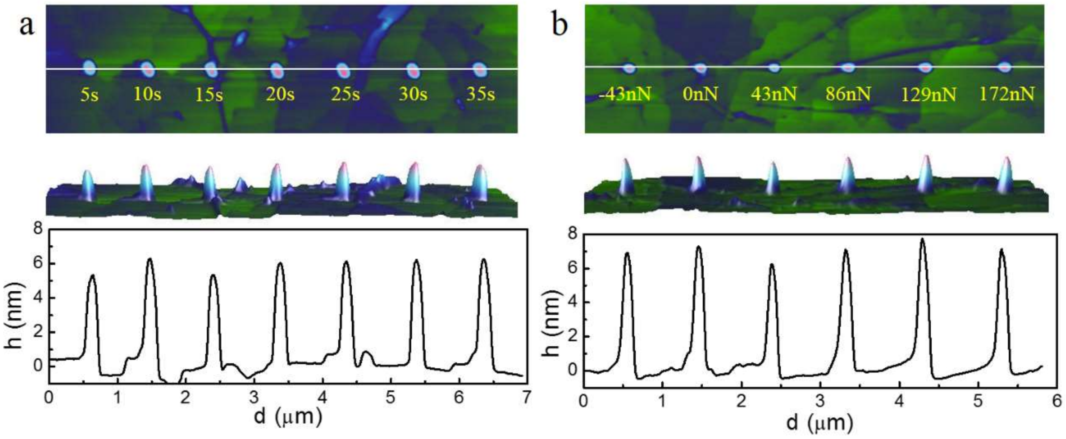

Supplementary Figure 5 | Graphene nanobubbles induced by different energized time and load pressure. a, AFM 2D image (top) and 3D image (middle) of GNBs formed by the energized time from 5 to 35 s with -5 V tip bias. The line profile alone the white line in top image is also presented (bottom). **b**, AFM 2D image (top) and 3D image (middle) of GNBs formed by the load from -43 to 172 nN with -6 V tip bias. The line profile alone the white line in top image is also displayed (bottom).

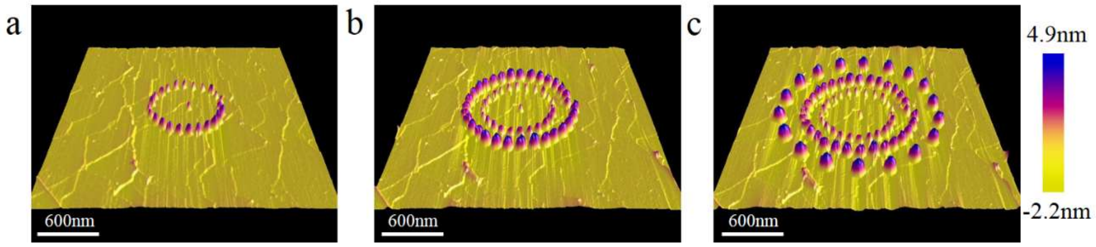

Supplementary Figure 6 | GNBs with "corral" pattern created by AFM tip using ramp mode. a, The first bubble corral and central bubble are created by the tip bias of -6 V. **b**, The second bubble corral is created by the tip bias of -7 V. **c**, The third bubble corral is created by the tip bias of -8 V.

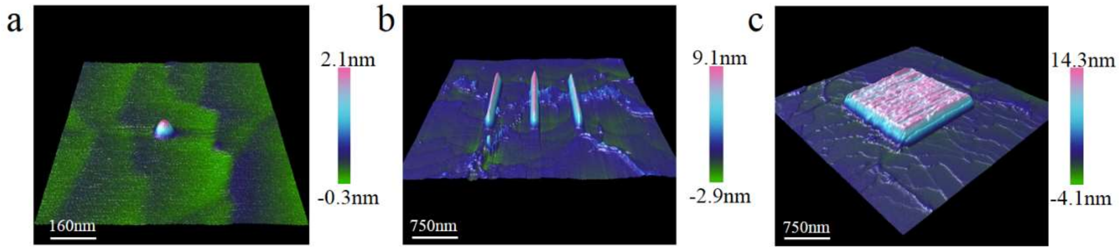

Supplementary Figure 7 | Graphene nanobubbles with diverse morphologies. a, Conventional circular graphene nanobubble. **b**, Linear graphene bubbles. **c**, Square graphene bubble.

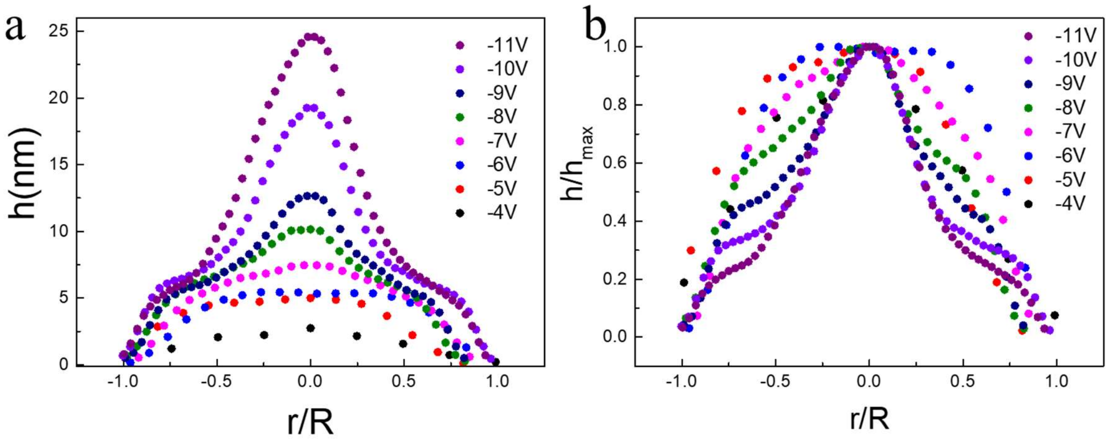

Supplementary Figure 8 | Normalized bubble profiles a, Normalized in-plane positions by the radii of GNBs created by AFM with various voltages. **b**, Normalized both in-plane positions by the radii and out-of-plane profiles by the maximum heights of GNBs created by AFM with various voltages.

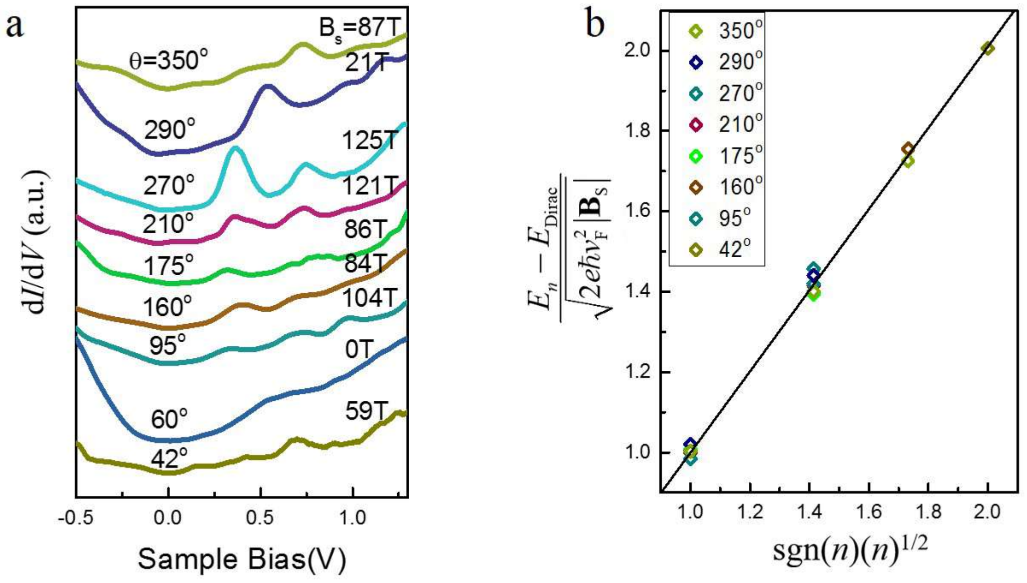

Supplementary Figure 9 | STS data collected at different rotational angle of the first GNB. a, dI/dV spectra recorded on the hollow regular triangles at different rotational angle in the inner circle (or the first circle line) on GNB shown in Fig. 3b. **b**, The energy of pseudo-Landau level peaks $(E_n - E_{\mathrm{Dirac}})/\sqrt{2e\hbar v_F^2|\mathbf{B}_S|}$ extracted from panel **a**, as a function of $\mathrm{sgn}(n)(n)^{1/2}$, following the linear fitting of equation (4). Source data are provided as a Source Data file.

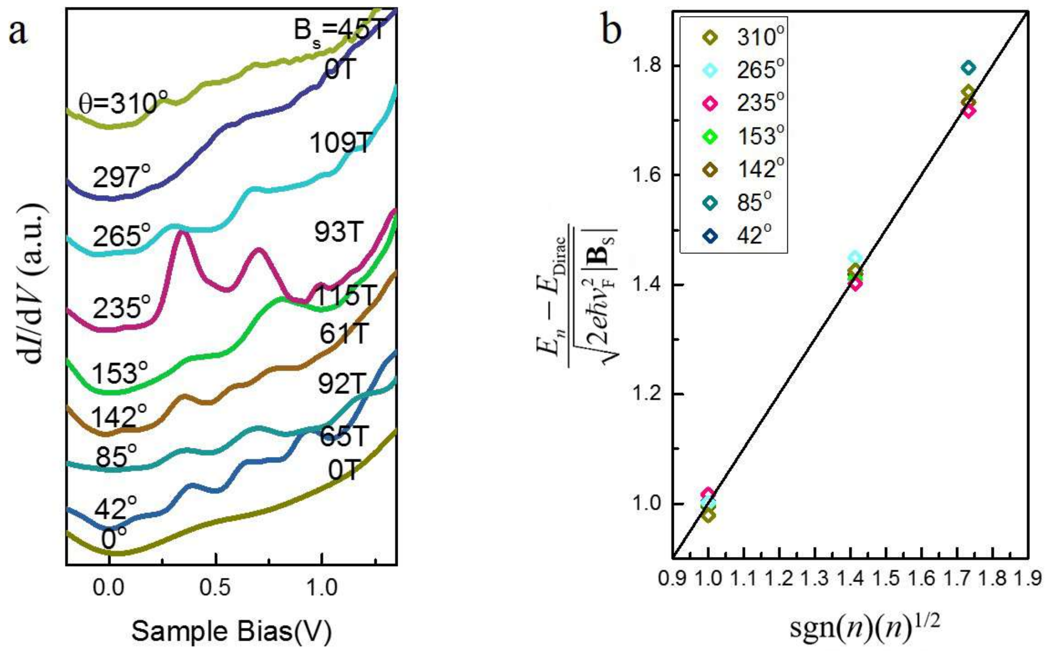

Supplementary Figure 10 | STS data collected at different rotational angle of the first GNB. **a**, *dI/dV* spectra recorded on the hollow inverted triangles at different rotational angle in the outer circle (or the third circle line) on GNB shown in Fig. 3b. **b**, The energy of pseudo-Landau level peaks $(E_n - E_{\mathrm{Dirac}})/\sqrt{2e\hbar v_F^2 |\mathbf{B}_S|}$ extracted from panel **a**, as a function of $\mathrm{sgn}(n)(n)^{1/2}$, following the linear fitting of equation (4). Source data are provided as a Source Data file.

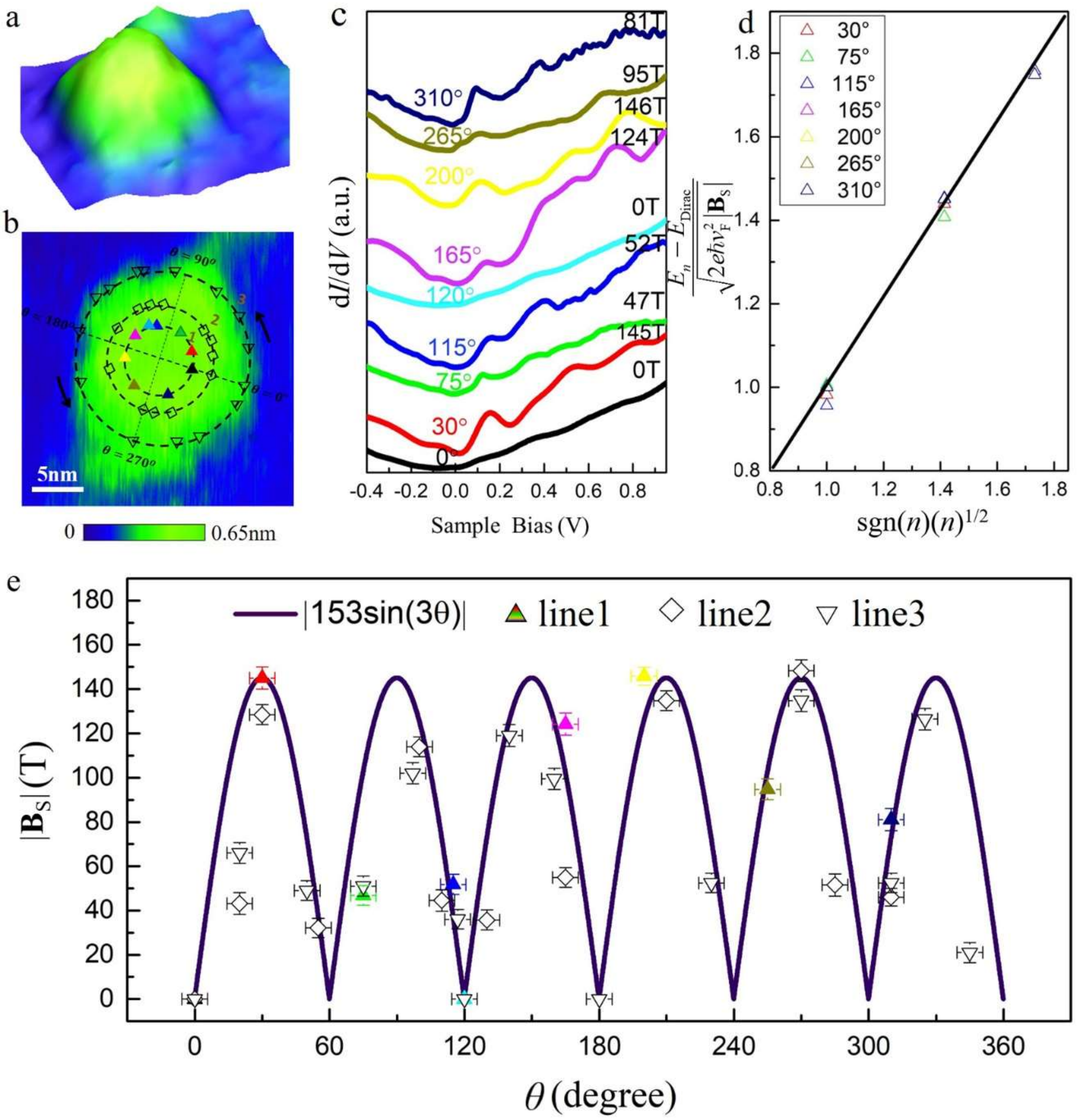

Supplementary Figure 11 | STM images and STS spectra of the second graphene bubble. a, 3D topographic image of a GNB with the basal radius of ~10nm and maximum height of ~ 0.65 nm (V_b = 500 mV, I_s = 0.2 nA). **b**, 2D projection of the GNB. **c**, *dI/dV* spectra recorded on the colored triangles at different rotational angle in the inner circle (or

the first circle line) on GNB shown in **b**. **d**, The energy of pseudo-Landau level peaks $\left(E_n - E_{\mathrm{Dirac}}\right)/\sqrt{2e\hbar v_{\mathrm{F}}^2\,|\mathbf{B}_{\mathrm{S}}|}$ extracted from panel c, as a function of $\mathrm{sgn}\left(n\right)\left(n\right)^{1/2}$. The solid line is a linear fit of the data with equation (4) in main text. **e**, The $|\mathbf{B}_{\mathrm{S}}|$ field as a function of the rotational angle θ. The data can be fitted by a six-fold-symmetric function as $|\mathbf{B}_{\mathrm{S}}| = |153\sin(3\theta)|$. Error bars represent the experimental uncertainties (s.d.) in determining the angles (in x axis) and extracted $|\mathbf{B}_{\mathrm{S}}|$ values (in y axis). Source data are provided as a Source Data file.

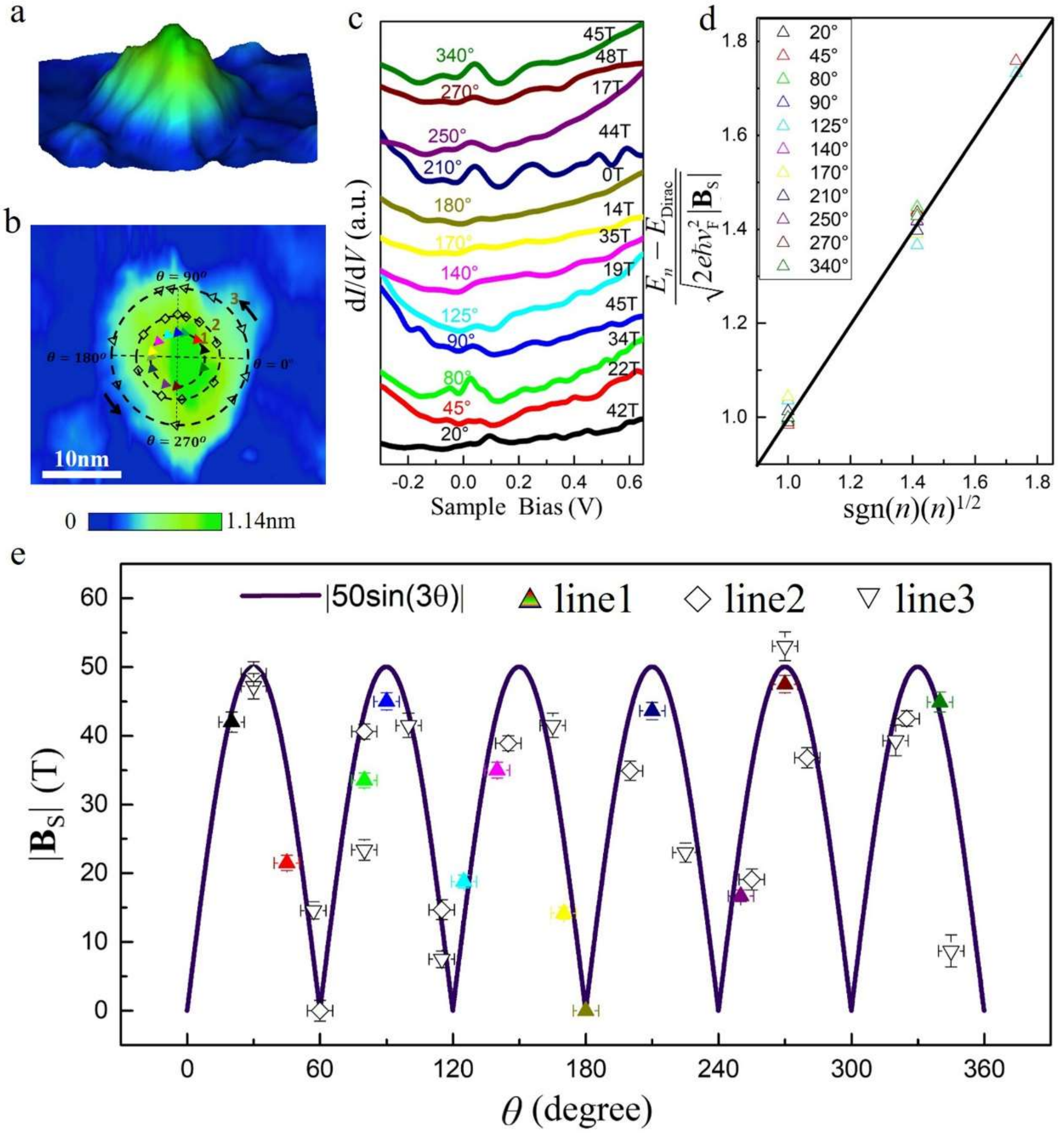

Supplementary Figure 12 | STM images and STS spectra of the third graphene bubble. **a**, 3D topographic image of a GNB with the basal radius of ~12.5nm and maximum height of ~1.14 nm (V_b = 800 mV, I_s = 0.2 nA). **b**, 2D projection of the GNB. **c**, d*I*/d*V* spectra recorded on the colored triangle at different rotational angle on GNB shown in **b**. **d**,

normalized peak energy $\left(E_n - E_{\mathrm{Dirac}}\right)/\sqrt{2e\hbar v_{\mathrm{F}}^2\left|\mathbf{B}_{\mathrm{S}}\right|}$ shows a linear dependence against $\mathrm{sgn}\left(n\right)\left(n\right)^{1/2}$. **e**, The $|\mathbf{B}_{\mathrm{S}}|$ field as a function of the rotational angle θ. The data can be fitted by a six-fold-symmetric function as $|\mathbf{B}_{\mathrm{S}}| = |50\sin(3\theta)|$. Error bars represent the experimental uncertainties (s.d.) in determining the angles (in x axis) and extracted $|\mathbf{B}_{\mathrm{S}}|$ values (in y axis). Source data are provided as a Source Data file.

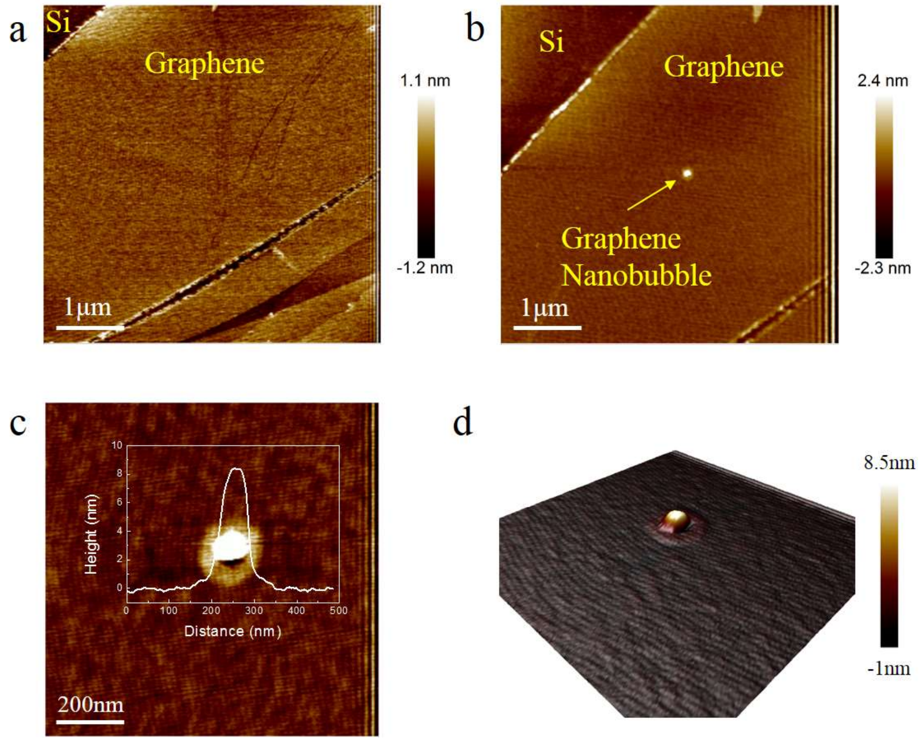

Supplementary Figure 13 | The AFM image of graphene nanobubble formed on hydrogen terminated Si substrate. a, The graphene transferred onto hydrogen-terminated Si substrate prior to the formation of graphene nanobubble. **b**, The graphene nanobubble on hydrogen-terminated Si substrate created by AFM tip with -6 V tip bias. **c**, The magnifying image of graphene nanobubble. **d**, The AFM 3D image of graphene nanobubble in **c**.

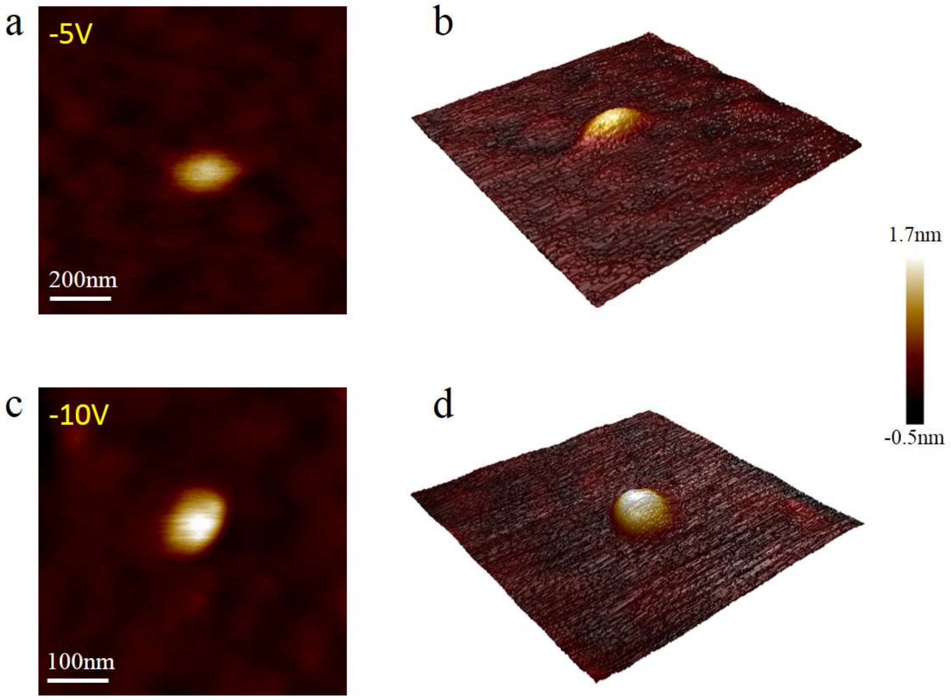

Supplementary Figure 14 | The AFM image of graphene nanobubbles formed on hydrogen terminated SiO_2/Si substrate. a, Graphene nanobubble created by AFM with -5 V tip bias. **b**, The AFM 3D image of graphene nanobubble in **a**. **c**, Graphene nanobubble created by AFM with -10 V tip bias. **d**, The AFM 3D image of graphene nanobubble in **c**.

Supplementary References

1. Lee, J.-H., Lee, E. K., Joo, W.-J., Jang, Y., Kim, B.-S., Lim, J. Y., Choi, S.-H., Ahn, S. J., Ahn, J. R. & Park, M.-H. Wafer-scale growth of single-crystal monolayer graphene on reusable hydrogen-terminated germanium. *Science* **344**, 286-289 (2014).
2. Tian, C. S. & Shen, Y. R. Structure and charging of hydrophobic material/water interfaces studied by phase-sensitive sum-frequency vibrational spectroscopy. *Proc. Natl. Acad. Sci. U.S.A.* **106**, 15148-15153 (2009).
3. Ji, N., Ostroverkhov, V., Chen, C.-Y. & Shen, Y.-R. Phase-sensitive sum-frequency vibrational spectroscopy and its application to studies of interfacial alkyl chains. *J. Am. Chem. Soc.* **129**, 10056-10057 (2007).
4. Zahler, J. M., Fontcuberta i Morral, A., Griggs, M. J., Atwater, H. A. & Chabal, Y. J. Role of hydrogen in hydrogen-induced layer exfoliation of germanium. *Phys. Rev. B* **75**, 035309 (2007).
5. Jobson, K. W., Wells, J. P. R., Schropp, R. E. I., Carder, D. A., Phillips, P. J. & Dijkhuis, J. I. Relaxation processes of the Ge–H stretch modes in hydrogenated amorphous germanium. *Phys. Rev. B* **73**, 155202 (2006).
6. Levy, N., Burke, S. A., Meaker, K. L., Panlasigui, M., Zettl, A., Guinea, F., Castro Neto, A. H. & Crommie, M. F. Strain-induced pseudo-magnetic fields greater than 300 tesla in graphene nanobubbles. *Science* **329**, 544-547 (2010).
7. Khestanova, E., Guinea, F., Fumagalli, L., Geim, A. K. & Grigorieva, I. V. Universal shape and pressure inside bubbles appearing in van der Waals heterostructures. *Nat.*

Commun. **7**, 12587 (2016).

8. Lu, J., Neto, A. H. & Loh, K. P. Transforming Moire blisters into geometric graphene nano-bubbles. *Nat. Commun* **3**, 823 (2012).
