# Supplementary material for: Programmable graphene nanobubbles with three-fold symmetric pseudo-magnetic fields
Source: Nat Commun. 2019 Jul 16;10:3127. doi: 10.1038/s41467-019-11038-7 (PMC6635427; doi:10.1038/s41467-019-11038-7)
Supplement: Supplementary file 2 — Description of Additional Supplementary Files [file 41467_2019_11038_MOESM2_ESM.pdf]

Description of Additional Supplementary Files

File Name: Supplementary Movie 1
Description: This movie shows the complete flow for the programmable GNBs fabricated by AFM using contact mode

File Name: Supplementary Movie 2
Description: This movie shows the complete flow for the programmable GNBs fabricated by AFM using ramp mode.
